# Mechanistic Insights into Peptide Binding and Deactivation of an Adhesion G Protein-Coupled Receptor

**DOI:** 10.3390/molecules29010164

**Published:** 2023-12-27

**Authors:** Victor A. Adediwura, Yinglong Miao

**Affiliations:** Department of Pharmacology and Computational Medicine Program, University of North Carolina at Chapel Hill, Chapel Hill, NC 27599, USA; vadediwu@ad.unc.edu

**Keywords:** adhesion G protein-coupled receptors, peptides, deactivation, Gaussian accelerated molecular dynamics (GaMD), drug design

## Abstract

Adhesion G protein-coupled receptors (ADGRGs) play critical roles in the reproductive, neurological, cardiovascular, and endocrine systems. In particular, ADGRG2 plays a significant role in Ewing sarcoma cell proliferation, parathyroid cell function, and male fertility. In 2022, a cryo-EM structure was reported for the active ADGRG2 bound by an optimized peptide agonist IP15 and the Gs protein. The IP15 peptide agonist was also modified to antagonists 4PH-E and 4PH-D with mutations of the 4PH residue to Glu and Asp, respectively. However, experimental structures of inactive antagonist-bound ADGRs remain to be resolved, and the activation mechanism of ADGRs such as ADGRG2 is poorly understood. Here, we applied Gaussian accelerated molecular dynamics (GaMD) simulations to probe conformational dynamics of the agonist- and antagonist-bound ADGRG2. By performing GaMD simulations, we were able to identify important low-energy conformations of ADGRG2 in the active, intermediate, and inactive states, as well as explore the binding conformations of each peptide. Moreover, our simulations revealed critical peptide-receptor residue interactions during the deactivation of ADGRG2. In conclusion, through GaMD simulations, we uncovered mechanistic insights into peptide (agonist and antagonist) binding and deactivation of the ADGRG2. These findings will potentially facilitate rational design of new peptide modulators of ADGRG2 and other ADGRs.

## 1. Introduction

G protein-coupled receptors (GPCRs) are essential for various physiological functions, including muscle contraction, hormone production, and neurotransmission [1]. Trafficking trajectories of the G protein and β-arrestin-mediated GPCR signaling pathways have been explored [2,3], providing a framework for drug design of GPCRs [4,5]. Adhesion GPCRs (ADGRs), which are class B2 GPCRs, perform a variety of roles in the endocrine, neurological, and immunological systems [6]. Regulation of numerous physiological elements, including the circulatory, endocrine, neurological, and reproductive systems, is greatly influenced by ADGRs [7,8,9,10,11,12]. In particular, ADGRG2 plays important physiological roles in the human body. Changes in ADGRG2 are linked to male infertility [13,14]. A loss-of-function mutation known as c.G118T:p.Glu40* in ADGRG2 leads to a medical condition called congenital bilateral absence of vas deferens [15]. This mutation causes an early end to the protein-making process in the third exon of ADGRG2 [15]. Patients who have this mutation were found to have near-zero ADGRG2 mRNA transcripts and protein in the proximal epididymal tissue [15]. In addition, ADGRG2 accelerates the growth and spread of Ewing sarcoma cells in vitro and in vivo [16] and as such, it is highly expressed in Ewing sarcomas compared to normal tissues and other sarcomas [16]. Knocking down ADGRG2 in Ewing sarcoma cell lines led to a reduction in tumor growth and metastasis [16]. The ADGRG2 gene also promotes tumor growth and is responsible for inducing placental growth factor and matrix metalloproteinase1 in Ewing sarcomas [16]. Furthermore, ADGRG2 is found to be highly expressed in cell lines from prostate cancer, non-small-cell lung cancer, and melanomas, while it is expressed at moderate to low levels in cell lines from brain, ovary, breast, and colon cancers [16]. It is also identified as a marker for a subgroup of medulloblastoma characterized by overactive WNT signaling [17]. Silencing ADGRG2 in the Hs578T and MDA-MB-231 cell lines decreases cell adhesion and migration [18]. Antibodies targeting ADGRG2 accumulate in bone and soft tissue sarcomas, but they do not accumulate in the epididymis in vivo [19]. ADGRG2 has exceptional characteristics as an immunotherapeutic target. Moreover, the antibody targeting ADGRG2 that was employed in this study is accessible commercially and presents the potential to be a valuable resource for delivering antibody-based therapeutics [19]. Therefore, ADGRG2 has emerged as an important target for drug development for bone cancers and male infertility.

ADGRs self-cleave at a GPCR proteolysis site into the N-terminal and C-terminal fragments (NTF and CTF). The NTF induces signaling in distant cellular environments [20]. Cell–cell adhesions with membrane proteins such as GPR124 and ADGRG1 elicit cell signaling [21,22,23]. Soluble ligands induce signaling by binding to the extracellular region of GPR126 [24,25,26]. Mechanical forces and separation of the NTF from the CTF cause downstream signaling [27,28,29,30,31,32,33,34]. The CTF comprises the seven transmembrane (7TM) helical domains, three intracellular and three extracellular loops (ICL, ECL), and a short extracellular “Stachel” sequence at the N-terminus. Various studies [35,36,37,38,39] showed that the Stachel sequence can activate ADGRs. Dissociation of NTF has the potential to uncover the Stachel sequence. This, in turn, enables binding of the Stachel sequence to the 7TM domains of ADGRs. The mechanism of activation has been explored for NTF-truncated ADGRs. Particularly, the NTF-truncated ADGRG2 exhibits stronger constitutive G protein coupling activity (specifically Gs and Gq coupling) than full-length ADGRG2 [40,41]. This suggests that NTF is a negative allosteric regulator of the CTF. Synthetic peptides that act as agonists and are similar to the Stachel sequence, such as the IP15 peptide agonist, were used to solve the cryo-EM of the full-length ADGRG2 with the Gαs, β, γ heterotrimer [40]. A mutant of ADGRG1 that lacked both NTF and Stachel was shown to maintain the constitutive activation of the nuclear factor of activated T cells and recruitment of β-arrestins [42]. This indicated that NTF has dual roles, i.e., it shields the Stachel peptide and inhibits the intrinsic activity of the CTF [43]. Moreover, studies have shown that ADGRG1, GPR64 (ADGRG2), ADGRG5, and ADGRG6 can be activated by tethered peptide agonists when NTF is removed due to autoproteolysis [30,35,44,45,46]

Previous studies demonstrated that the endogenous peptide agonist p15, with sequence “TSFGILLDLSRTSLP”, activates ADGRG2 for downstream Gs, Gq, and G12/13 signaling [45,46]. Notably, p15 does not influence or regulate other ADGRs, such as GPR110 and GPR133, and thus specifically targets the ADGRG2 receptor [46]. However, the usage of p15 for characterizing ADGRG2 is limited by its low affinity. In order to address this issue, Sun et al. conducted biochemical experiments, such as alanine scanning mutagenesis, to develop an optimized agonist called VPM-15. The first residue in the Stachel sequence, which is threonine (T), was mutated to valine (V), and the third residue, phenylalanine (F), was mutated to 4-methyl phenylalanine (4PH) to produce an optimized agonist with sequence “VS4PHGILLDLSRTSLP” [45]. The optimized peptide agonist called VPM-p15 could activate the ADGRG2 and significantly improved the binding affinity by more than two orders of magnitude compared to the endogenous peptide agonist p15 [45]. This agonist was used to investigate the potential site where critical residues responsible for ADGRG2 activity induced by VPM-15 are located. ECL2 and TM6 were critical for activation of ADGRG2 induced by VPM-15 binding. Gad et al. further delineated key residues in the ECL2 and TM6 that play key roles in ADGRG2 activation by the Stachel sequence. They aligned the ECL2 residues of 33 human ADGRs obtained from GPCRdb or Uniprot. Most of the ADGRs had an aliphatic residue, such as leucine or isoleucine, after a conserved CW motif (C778^ECL2^ and W779^ECL2^) in ADGRG2. Using site-directed mutagenesis, Gad et al. created mutants of the conserved residues (C778^ECL2^, W779^ECL2^, I780^ECL2^) in the ECL2. They showed that tryptophan and isoleucine were crucial for receptor stability and surface expression in HEK293 cells. Mutations of W779^ECL2^ and I780^ECL2^ to alanine eliminated the Stachel-mediated activation of ADGRG2 [43]

Recently, Xiao et al. replaced the first threonine of the VPM-15 with isoleucine, thereby generating a new optimized peptide agonist IP15. This modification showed a 10,000-fold increase in the peptide binding affinity compared with VPM-p15. Furthermore, this modification allowed them to solve the cryo-EM structure of ADGRG2 in full length in complex with the IP15 agonist and Gs protein at 3.1 Å resolution [40]. The Stachel sequence (p15) consists of two parts: the upper rim and lower rim. The lower rim is primarily hydrophobic and consists of “TSFGILLDL - - - - LP”. The p15 consensus motif of the consensus sequence FXφφφXφ interacts with the ADGRG2 hydrophobic residues located at the TM1 to TM7 regions of the orthosteric pocket [40]. The upper rim of the p15 agonist consists of the residues “- - - - - - - SRTS - -”, which are hydrophilic. Biochemical experiments support the proposition that these hydrophilic residues stabilize the orientation and overall configuration of p15 in the orthosteric pocket [40]. The IP15 agonists’ last four residues, “TSLP”, were not resolved in the cryo-EM structure. However, to explore the potential role of these residues in receptor stabilization, we included them in our simulation as they contain the hydrophilic residues “TS”. Furthermore, to generate peptide antagonists, Xiao et al. mutated the 4PH in the IP15 peptide agonist to Asp and Glu acid, respectively. These mutations changed the functional role of the peptide from an agonist to antagonist. However, the inactive antagonist-bound structure of the ADGRG2 receptor is unavailable, despite the peptide antagonists being identified biochemically. The mechanism of ADGRG2 deactivation remains elusive.

Gaussian accelerated molecular dynamics (GaMD) is a computational technique that provides unconstrained enhanced sampling and free-energy calculation of biomolecules without constraints [47]. It adds a harmonic boost potential to lower the system energy barriers and accelerate biomolecular simulations. Because the boost potential usually exhibits Gaussian distribution, GaMD simulations can be properly reweighted using cumulant expansion to the second. GaMD has proven useful in capturing rare and complex events such as ligand binding [47,48,49,50,51,52,53,54], protein–protein/membrane/nucleic acid interactions[55,56,57,58,59,60], protein folding and unfolding[50], GPCR activation[48], GPCR allostery [61], and other system dynamics [62,63,64,65,66,67].

Here, we applied GaMD simulations to elucidate mechanistic insights into deactivation of the ADGRG2 and examine its different conformational states. We computationally mutated 4PH residue in the IP15 peptide agonist to Asp and Glu to generate the antagonist systems. We applied GaMD to refine the peptide-receptor complex structures, examined dynamic interactions between the peptides and ADGRG2, and captured antagonist-induced deactivation of ADGRG2. For specific reaction coordinates, energy profiles were computed via reweighting of the GaMD simulations.

## 2. Results

### 2.1. Agonist IP15-Gs-Bound ADGRG2 Sampled Only the “Active” State

**Figure 1:**
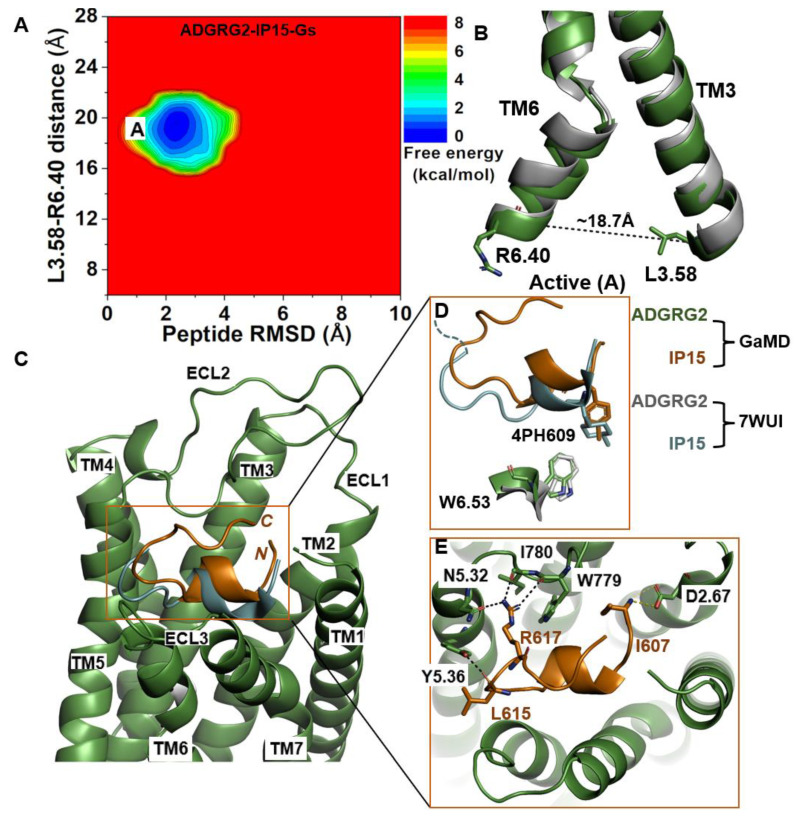
**Agonist IP15-Gs-bound ADGRG2 sampled only the “Active” (“A”) state.** (**A**) Free-energy profile of the ADGRG2–IP15-Gs complex calculated from GaMD simulations focused on the distance between the Cα atoms of L^3.58^-R^6.40^ and peptide RMSD relative to the cryo-EM structure (PDB:7WUI). (**B**) Alignment of the “Active” low-energy conformation (green) and the 7WUI cryo-EM structure of ADGRG2 (gray). The L^3.58^-R^6.40^ distance in the active conformation is ~18.7 Å. (**C**) Top-ranked structural cluster of the IP15 agonist obtained from GaMD simulations (orange) compared with the cryo-EM conformation (cyan). (**D**) Orientation of the modified residue (4PH) in IP15 and the rotameric toggle switch W^6.53^ in ADGRG2 as depicted by sticks. The cyan dashed line represents the last four residues (TSLP) that were not solved in the cryo-EM structure. (**E**) Critical interactions at the atomistic level between the peptide (orange sticks) and receptor (green sticks) observed in the GaMD simulations. The peptide agonist mediates hydrogen-bonding (black dash lines) and salt-bridge (yellow dash lines) interactions with receptor residues W779^ECL2^, I780^ECL2^, D^2.67^, Y^5.36^, and N^5.32^, which were experimentally shown to be critical for activation of ADGRG2.

We calculated the 2D free-energy profile of the IP15-Gs-bound ADGRG2 system using the distance of the Cα atoms of L^3.58^-R^6.40^ and the peptide RMSD relative to the cryo-EM structure (PDB:7WUI) as reaction coordinates (Figure 1A). The amino acid residues in the TM helices of ADGRG2 are numbered using the Ballesteros–Weinstein scheme, in which the most conserved residue in helix I is assigned I.50 and the others are numbered decreasingly towards the N-terminus and increasingly towards the C-terminus [68]. Numbering in GPCRdb https://gpcrdb.org/protein/agrg2_human (accessed on 18 December 2023) [69] is used for other residues. A single low-energy state (“active”) was identified from the free-energy profile, in which the L^3.58^-R^6.40^ distance exhibited a local minimum at ~18.7 Å (Figure 1B). The low-energy conformation of the rotameric toggle switch residue W^6.53^ obtained from structural clustering of GaMD simulations aligned perfectly with the cryo-EM structure (Figure 1D), indicating that the activation hub of ADGRG2 was not perturbed in GaMD simulations (Figure 1D).

In the GaMD simulations, we identified that key residues at the upper rim of the orthosteric pocket play important roles in defining the orientation and binding conformation of the agonist peptide. These interactions were also observed in the cryo-EM structure. The side-chain atoms NH2 and NH1 of residue R617 in IP15 formed hydrogen bonds with the side-chain atom ND2 of N^5.32^, the backbone O atom of I780^ECL2^, and the backbone O atom of W779^ECL2^ at distances of ~2.7 Å, ~2.9 Å, and ~2.8 Å, respectively. The backbone O atom of L615 in IP15 formed a hydrogen bond with Y^5.36^ (OH) at ~2.6 Å distance (Figure 1E). Moreover, the N-terminal end of the IP15 with residue I607 formed a salt-bridge interaction with the side chain of D^2.67^ (Figure 1E). Previous studies experimentally validated I780^ECL2^ and W779^ECL2^ residues in ECL2 to be critical for the activation of ADGRG2 by the cognate Stachel sequence [43]. They found that the p15 peptide agonist could not activate the ADGRG2 receptor when I780^ECL2^ and W779^ECL2^ were mutated to alanine.

### 2.2. Agonist IP15-Bound ADGRG2 without Gs Sampled the “Active” and “Intermediate” (“I1”) States

**Figure 2:**
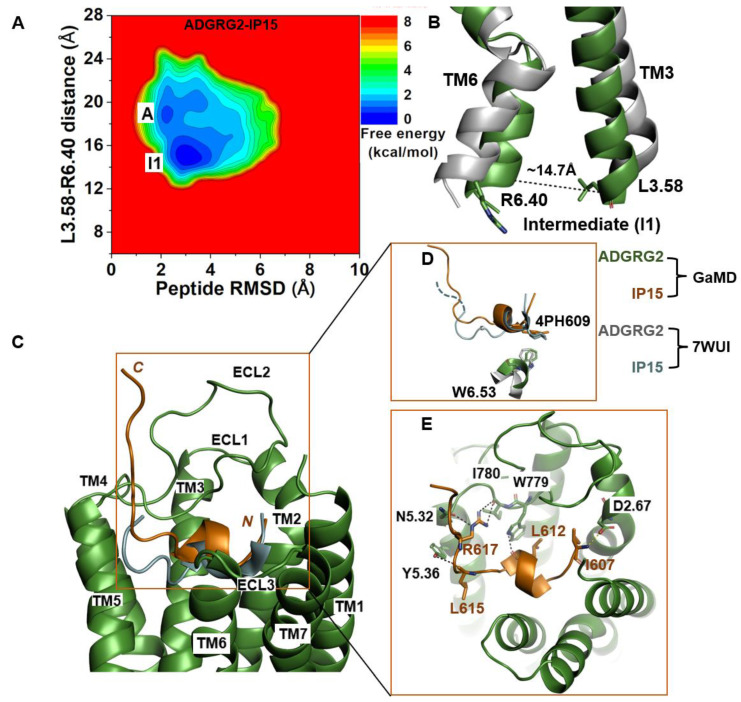
**Agonist IP15-bound ADGRG2 without Gs sampled the “Active” and “Intermediate 1” (“I1”) states.** (**A**) Free-energy profile of the ADGRG2–IP15 complex calculated from GaMD simulations focused on the distance between the Cα atoms of L^3.58^-R^6.40^ and peptide RMSD relative to the cryo-EM structure (PDB:7WUI). (**B**) Alignment of the intermediate “I1” low-energy conformation (green) with the 7WUI cryo-EM structure of ADGRG2 (gray). The L^3.58^-R^6.40^ distance in the intermediate conformation is ~14.7 Å. (**C**) Top-ranked structural cluster of the optimized agonist (IP15) obtained from GaMD simulations (orange) compared with the cryo-EM conformation (cyan). (**D**) Orientation of the modified residue 4PH in IP15 and the rotameric toggle switch W^6.53^ in ADGRG2 were depicted as sticks. The cyan dashed line represents the last four residues (TSLP) that were not solved in the cryo-EM structure. (**E**) Critical interactions at the atomistic level between the peptide (orange sticks) and receptor (green sticks) observed in the GaMD simulations. In this system, the peptide agonist mediates hydrogen-bonding interactions with receptor residues W779^ECL2^, I780 ^ECL2^, Y^5.36^, and N^5.32^ (black dashed lines) and salt-bridge interactions with D^2.76^ (yellow dashed lines).

The 2D free-energy profile of the IP15-bound ADGRG2 was calculated using the distance of the Cα atoms of L^3.58^-R^6.40^ distance and peptide RMSD relative to the cryo-EM structure (PDB:7WUI) as reaction coordinates. In the absence of the Gs, the IP15-bound ADGRG2 could transition from the “active” state to the “intermediate I1” state (Figure 2A). The distance of the Cα atoms of L^3.58^-R^6.40^ decreased to ~14.7 Å (Figure 2B). In the intermediate structure, the rotameric toggle switch W^6.53^ side chain tilted by ~15° from the cryo-EM structure (Figure 2D). The low-energy conformation obtained from the ADGRG2–IP15-Gs system showed that the W^6.53^ side chain perfectly aligned with the side chain of W^6.53^ of the cryo-EM structure of ADGRG2 (7WUI). However, in the ADGRG2–IP15 system, the W^6.53^ side chain tilted relative to the cryo-EM structure of ADGRG2 (7WUI). The χ^2^ torsional angle of the rotameric toggle switch W^6.53^ was measured to be 107.7° in the cryo-EM structure. The χ^2^ torsional angle of W^6.53^ in the GaMD simulation of the ADGRG2–IP15-Gs system was measured to be ~101.5°, indicating that the Gs-bound ADGRG2 maintained the cryo-EM structure. In the ADGRG2–IP15 system, the χ_2_ angle of W^6.53^ increased to 122.3°, suggesting that the W^6.53^ indole’s ring adopted a different orientation. Overall, the perturbation contributed to the conformational transition of ADGRG2 from the active to the intermediate I1 state (Figure 2D).

The top-ranked structural cluster of the IP15 agonist peptide in the ADGRG2–IP15 system showed an overall different conformation from that in the ADGRG2–IP15-Gs system, while the IP15 formed similar polar interactions as described for the ADGRG2–IP15-Gs system (Figure 1E and Figure 2E). Previous studies experimentally showed that mutations of residues I780^ECL2^ and W779^ECL2^ in ECL2 and W^6.53^ in TM6 abolished the binding of a peptide agonist to the ADGRG2 [43,45]. In the ADGRG2–IP15 system, the side-chain atoms NH2, NH1, and NE of R617 in IP15 formed hydrogen-bonding interactions with backbone O atoms of I780^ECL2^ and N^5.32^. The backbone O atom of L612 formed a hydrogen bond with the side-chain NE atom of W779^ECL2^ at ~3.0 Å distance. The backbone O atom of L615 in IP15 formed a hydrogen bond with Y^5.36^ (side-chain OH atom) at ~2.8 Å distance (Figure 2E). Moreover, the N-terminal residue I607 of the IP15 formed a salt-bridge interaction with side-chain atoms of D^2.67^ (Figure 2E).

### 2.3. Antagonist 4PH-E-Bound ADGRG2 System Sampled the “A”, “I1”, and “Inactive” (“IN”) States

**Figure 3:**
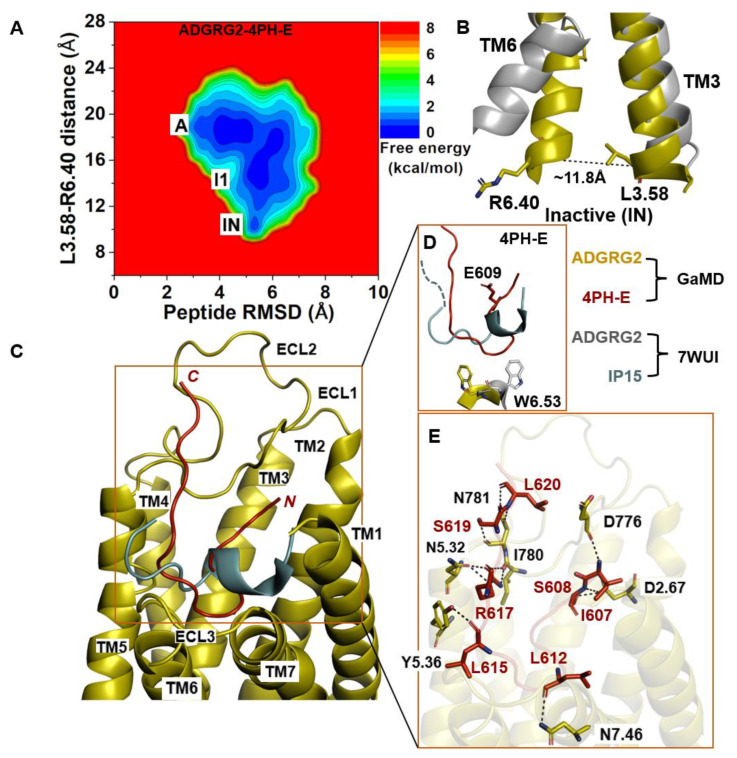
**Antagonist 4PH-E-bound ADGRG2 system sampled the “Active”, “Intermediate 1”, and “Inactive” (“IN”) states.** (**A**) Free-energy profile of the ADGRG2–4PH-E complex calculated from GaMD simulations focused on the distance between the Cα atoms of L^3.58^-R^6.40^ and peptide RMSD relative to the cryo-EM structure (PDB:7WUI). (**B**) Alignment of the “inactive” low-energy conformation (yellow) with the 7WUI cryo-EM structure of ADGRG2 (gray). The L^3.58^-R^6.40^ distance in the inactive low-energy conformation is ~13.8 Å. (**C**) Top-ranked structural cluster of the antagonist obtained from GaMD simulations (red) compared with the cryo-EM conformation (cyan). (**D**) Orientation of the modified antagonist residue E609 and the ADGRG2 rotameric toggle switch W^6.53^. The indole ring of W^6.53^ (yellow sticks) could flip by ~250° degrees relative to the cryo-EM structure. The cyan dashed line represents the last four residues (TSLP) that were not solved in the cryo-EM structure. (**E**) Critical interactions at the atomistic level between the antagonist peptide (red sticks) and receptor (yellow sticks) observed in the GaMD simulations. The peptide antagonist mediates hydrogen-bonding interactions with receptor residues N^5.32^, N781 ^ECL2^, I780^ECL2^, Y^5.36^, N^7.46^, D776^ECL2^, and D^2.67^ (black dashed lines).

The 2D free-energy profile of 4PH-E-bound ADGRG2 was plotted by using the distance of the Cα atoms L^3.58^ and R^6.40^ and the peptide RMSD relative to the cryo-EM structure (PDB:7WUI) as reaction coordinates (Figure 3A). The “inactive” state of the 4PH-E-bound ADGRG2 was sampled (Figure 3A). This observation was consistent with the experimental work performed by Xiao et al. describing this antagonist as much weaker than the 4PH-D antagonist. The distance of the Cα atoms of L^3.58^-R^6.40^ decreased to ~11.8 Å in the “inactive” state (Figure 3B). The “inactive” state obtained from structural clustering shows that the indole ring of the rotameric toggle switch W^6.53^ side chain dramatically tilted away from that of the cryo-EM structure (PDB:7WUI). The χ_2_ torsional angle of the W^6.53^ was measured to be −143.9°, accounting for a larger angular deviation from the χ_2_ torsional angle of the cryo-EM structure (PDB:7WUI) measured at 107.7°. The significant perturbation of the indole ring in the rotameric toggle switch W^6.53^ could be responsible for the receptor (ADGRG2) switch from the “intermediate I1” state to the “inactive” (Figure 3D). The pronounced perturbation that occurred at the toggle switch arose when 4-methyl-phenyalanine was mutated to glutamic acid (E609). The side chain of the glutamic acid residue (E609) became solvent when exposed by facing upward (Figure 3D). Such a different conformation of the peptide thus prompted the receptor residue W^6.53^ to seek alternative interaction partners with TM5 residues. The side chain of the receptor W^6.53^ formed hydrophobic interactions with residues F^5.43^, C^5.44^, and F^5.47^.

In the 4PH-E peptide, backbone N and O atoms of residue L620 and the side-chain OG atom of residue S619 formed hydrogen bonds with the side-chain OD1, ND2 atoms, and the backbone O atom of N781^ECL2^ at distances of ~3.3 Å, ~2.9 Å, and ~3.0 Å, respectively (Figure 3E). Moreover, the side-chain NH1 and NH2 atoms of R617 formed hydrogen bonds with the backbone O atom of I780^ECL2^ at ~2.9 Å and ~3.0 Å, respectively. The side-chain NE atom of R617 formed hydrogen-bonding interactions with the side-chain OD1 atom of N^5.32^ at ~2.8 Å distance. Furthermore, the backbone O atoms of L615 and L612 in the 4PH-E peptide formed hydrogen-bonding interactions with the side-chain OH atom of Y^5.36^ and the side-chain ND2 atom of N^7.46^. The side-chain OG atom and backbone N atom of S608 formed a hydrogen-bonding interaction with the side-chain OD1 atom of D^2.67^. The N-terminal residue I607 of 4PH-E formed a hydrogen-bonding interaction with the backbone O atom of D776^ECL2^ (Figure 3E).

### 2.4. Antagonist 4PH-D-Bound ADGRG2 Sampled the “A”, “I1”, and “IN/Bound” “IN/Lifted” States

**Figure 4:**
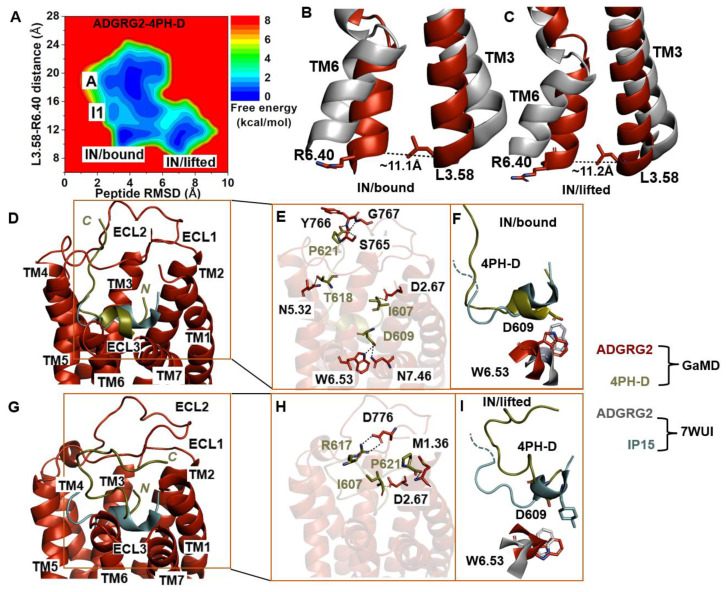
Antagonist 4PH-D-bound ADGRG2 sampled “Active”, “Intermediate 1 (I1)”, “Inactive/bound” (“IN/bound”), and “Inactive/lifted” (“IN/lifted”) states. (**A**) Free-energy profile of the ADGRG2–4PH-D complex calculated from GaMD simulations focused on the distance between the Cα atoms of L^3.58^-R^6.40^ and peptide RMSD relative to the cryo-EM structure (PDB:7WUI). (**B**,**C**) Alignment of the “IN/bound” and “IN/lifted” low-energy conformations (red) with the 7WUI cryo-EM structure of ADGRG2 (gray). The L^3.58^-R^6.40^ distance in the “IN/bound” and “IN/lifted” low-energy conformations are ~11.1 Å and ~11.2 Å, respectively. (**D**) Top-ranked structural cluster of the IN/bound state obtained from GaMD simulations compared with the cryo-EM structure (cyan). (**E**) Critical interactions at the atomistic level between the “IN/bound” antagonist peptide and receptor observed in the GaMD simulations. In the “IN/bound” state, the peptide antagonist mediates salt-bridge interactions with receptor residues D^2.67^ and hydrogen-bonding interactions with N^7.46^, W^6.53^, N^5.32^, S765 ^ECL2^, Y766 ^ECL2^, and G767 ^ECL2^ (black dashed lines). (**F**) Orientation of the modified antagonist residue D609 in the “IN/bound” state and the ADGRG2 rotameric toggle switch W^6.53^. The cyan dashed lines represent the last four residues (TSLP) that were not solved in the cryo-EM structure. (**G**) Top-ranked structural cluster of the IN/lifted state obtained from GaMD simulations compared with the cryo-EM structure (cyan). (**H**) Critical interactions at the atomistic level between the “IN/lifted” antagonist peptide and receptor observed in the GaMD simulations. In the “IN/lifted” state, the peptide antagonist mediates hydrogen-bonding interactions with receptor residues M^1.36^ and D776 ^ECL2^ (black dashed lines) and salt-bridge interactions with D^2.67^ (yellow dashed lines). (**I**) Orientation of the modified antagonist residue D609 in the “IN/lifted” state and the rotameric toggle switch of ADGRG2 W^6.53^. The cyan dashed lines represent the last four residues (TSLP) that were not solved in the cryo-EM structure.

Further GaMD simulations of the ADGRG2–4PH-D system revealed two distinct low-energy states (“IN/bound” and “IN/lifted”) of the ADGRG2 receptor (Figure 4A). The distance of the Cα atoms of L^3.58^-R^6.40^ in these two states were ~11.1 Å and ~11.2 Å, respectively (Figure 4B,C). The peptide RMSD relative to the cryo-EM conformation in the “IN/bound” and “IN/lifted” states were ~3.9 Å and ~7.0 Å, respectively (Supplementary Figure S2D).

The low-energy conformation of the 4PH-D in the “IN/bound” state revealed essential polar interactions with the ADGRG2 receptor. In the 4PH-D peptide, the backbone OT1 and OT2 atoms of residue P621 formed hydrogen-bonding interactions with the side-chain OG atom and backbone N atoms of S765^ECL2^, Y766^ECL2^, and G767^ECL2^. Furthermore, the side-chain OG1, OD1, and OD2 atoms of residues T618 and D609 in the 4PH-D peptide antagonist formed hydrogen-bonding interactions with the side-chain OD1, NE1, and ND2 atoms of N^5.32^, W^6.53^, and N^7.46^ (Figure 4E). The N-terminal residue I607 of the 4PH-D peptide formed a salt bridge with the side-chain OD1 atom of D^2.67^.

We observed the formation of a hydrogen bond between the NE1 atom of the indole ring in receptor W^6.53^ and the antagonist peptide residue D609. In the cryo-EM structure, the agonist peptide residue 4PH interacted hydrophobically and established packing contacts with W^6.53^. For the two residues W^6.53^ and D609, to establish hydrogen-bonding interactions, the indole ring of W^6.53^ must undergo a ~180° rotation, suggesting that the indole ring flipped upward to mediate polar interaction with D609 of the 4PH-D antagonist. The χ_2_ torsional angle of the rotameric toggle switch W^6.53^ in ADGRG2–4PH-D was measured at −81.9°. The torsion angular difference between the antagonist 4PH-D-bound ADGRG2 and the cryo-EM structure is about ~188°. In the two ADGRG2–4PH-E and ADGRG2–4PH-D systems, W^6.53^ rotated by ~250° and ~188°, respectively. A small degree of ~15° rotation of W^6.53^ was observed in the ADGRG2–IP15 system where it sampled the intermediate I1 state of ADGRG2. Our simulations further reinforced the role of the toggle switch in GPCR biology, particularly in the context of the ADGRG2 receptor.

In the “IN/lifted” state, the peptide antagonist mediated polar interactions with the ADGRG2 receptor. The side-chain NH1 and NH2 atoms of residue R617 and the side-chain OT1 atom of residue P621 in the 4PH-D formed hydrogen-bonding interactions with the side-chain OD1, OD2 atoms of D776^ECL2^ and the backbone N atom of M^1.36^. Moreover, the charged N-terminal residue I607 of the peptide antagonist formed a salt-bridge interaction with the side chain of D^2.67^ (Figure 4H).

## 3. Discussion

In this study, we adopted a recently solved cryo-EM structure of the ADGRG2–IP15-Gs complex PDB 7WUI [40] for extensive all-atom simulations using the GaMD method, which revealed distinct binding conformations of antagonist peptides and deactivation of ADGRG2. A comparison of interactions of agonists and antagonists with the receptor allowed us to identify important residues during receptor deactivation. The two agonist-bound systems exhibited similar polar interactions with IP15. In all the systems, the peptide mediated hydrogen-bonding interactions with N^5.32^ and salt-bridge/hydrogen-bonding interactions with D^2.67^ (Table 1). We hypothesized that these residues served as an anchor that stabilized the respective peptide in the receptor pocket. The low-energy conformations of the IP15 agonist obtained from our simulations agreed with the cryo-EM structure and biochemical experiments. For example, biochemical experiments showed that residue mutations in ECL2 (I780^ECL2^ and W779^ECL2^) and TM6 (W^6.53^) abolished activation by the cognate Stachel peptide agonist in ADGRG2 [43,45]. In our GaMD simulations, we revealed the corresponding hydrogen-bonding interactions between IP15 and the orthosteric pocket of ADGRG2 residues I780^ECL2^, W779^ECL2^, Y^5.36^, and N^5.32^ (Figure 1E and Figure 2E). Such interactions could be critical to stabilize binding conformation of the IP15 agonist, being consistent with the biochemical analysis [40].

In class A GPCRs, the distance between the TM3 and TM6 intracellular domains of the inactive conformation is shorter compared to that of the active states. For example, in the β2-adrenergic receptor, the TM3–TM6 distance decreases during the transition from the active to the inactive state [70,71,72]. Here, we observed that the TM3-TM6 distance of the antagonist-bound ADGRG2 decreased. In the cryo-EM structure of active ADGRG2 (PDB:7WUI), the L^3.58^-R^6.40^ distance was ~19.9 Å. In the ADGRG2–4PH-E and ADGRG2–4PH-D antagonist-bound systems, the L^3.58^-R^6.40^ distance decreased to ~11.8 Å and ~11.1 Å, respectively. During GPCR activation, the TM6 intracellular domain undergoes significant outward movement as facilitated by the highly flexible ICL3, which is usually accompanied by inward movement of the NPxxY motif in the TM7 intracellular domain [70,72].

The CWI (C778^ECL2^, W779^ECL2^, and I780^ECL2^) motif [43] in ECL2 played a pivotal role in receptor activation be mediating hydrogen-bonding interactions with the agonist peptide. In the ADGRG2–4PH-D system, we identified SYG residues (S765^ECL2^, Y766^ECL2^, and G767^ECL2^). The SYG residues were in proximity to and directly above the CWI residues within the ECL2 (Figure 4E). The SYG residues appeared to play an important role in the receptor deactivation. The SYG residues counterbalanced the CWI residues by interacting with the antagonist peptide, specifically the negatively charged terminal carboxylic group of P621 (4PH-D antagonist), thereby inducing the receptor’s inactive state. This intricate spatial and temporal interplay could ensure a delicate equilibrium in receptor function, allowing swift toggling between active and inactive states. These insights obtained from our simulations will require a series of experimental studies for validation. Aside from the rotameric toggle switch W^6.53^, residues that could be relevant in ADGRG2 deactivation also included N^7.46^, D776^ECL2^, and N781^ECL2^.

Aside from the CWI and SYG interplay in receptor deactivation, the toggle switch plays a crucial role in deactivation of the ADGRG2 receptor [45]. In the antagonist ADGRG2–4PH-E and ADGRG2–4PH-D systems, the indole ring of the rotameric toggle switch W^6.53^ could rotate by ~250° and 180°, respectively, relative to the cryo-EM structure of ADGRG2 (PDB:7WUI) (Figure 3D and Figure 4F). In the intermediate I1 state of ADGRG2–IP15, the indole ring of W^6.53^ rotated by ~15° (Figure 2D). However, in the ADGRG2–IP15-Gs system, the W^6.53^ indole ring aligned well with the cryo-EM structure. Thus, the differential conformations of the rotameric toggle switch W^6.53^ appeared to dictate the receptor “active”, “inactive”, and “intermediate” states.

The ADGRG2–4PH-E system faintly sampled the inactive state (Figure 3A, Supplementary Figure S3C). In comparison, the 4PH-D-bound ADGRG2 system broadly sampled two conformations of the inactive state we dubbed “IN/bound” and “IN/lifted”. In the “IN/bound” state, the peptide antagonist (4PH-D) bound stably in the pocket while the “IN/lifted” state showed upward movement of the peptide with ~7.0 Å RMSD relative to the cryo-EM structure (Figure 4A, Supplementary Figure S2D). The GaMD simulation findings were highly consistent with the experimental data that the 4PH-D antagonist inhibited the cAMP accumulation much better than the 4PH-E antagonist [40].

It is worth mentioning that ECL2 is highly flexible in ADGRG2, even in the active agonist-Gs-bound structure [40]. In this regard, the experimental structure of the apo ADGRG2 is not available, for which ECL2 is expected to be flexible with likely multiple conformations. On the other hand, binding of antagonist peptides could stabilize ECL2 in certain conformations as shown in our GaMD simulations. This could be a plausible mechanism in the deactivation of ADGRG2. In summary, we uncovered a plausible mechanism by which antagonists 4PH-E and 4PH-D bind and deactivate the ADGRG2 receptor using GaMD simulations. The understanding we gained regarding ADGRG2 and peptide structural changes could offer a valuable foundation for the design and development of novel peptide regulators of ADGRG2 and other ADGRs.

## 4. Materials and Methods

### 4.1. Gaussian Accelerated Molecular Dynamics (GaMD)

GaMD is an enhanced sampling MD technique that adds a harmonic boost potential to smoothen the potential energy surface of biomolecules, thereby reducing energy barriers [47]. GaMD is a well-established enhanced sampling MD simulation method, and its theory is well described in [47].

### 4.2. Simulation Protocol

The cryo-EM structure of active IP15-Gs-bound ADGRG2 (PDB:7WUI) at 3.1 Å resolution was used to set up the simulation systems. The 7WUI structure includes the Gs protein (Gα, Gβ, and Gγ), ADGRG2, and optimized agonist IP15. Missing residues in the extracellular loop 2 (ECL2), extracellular loop 3 (ECL3), intracellular loop 3 (ICL3), and Gα subunit were added accordingly using the Swiss Modeler. The last four residues missing from the IP15 agonist (TSLP) were added by copying their coordinates from the 7WUQ PDB structure after aligning the peptides. This new construct served as our control system of the active ADGRG2–IP15-Gs complex. We then removed the G protein to obtain only the agonist IP15-bound ADGRG2. To generate the antagonist-bound ADGRG2 systems (ADGRG2–4PH-D and ADGRG2–4PH-E), we used UCSF chimera [73] to mutate the 4-methyl phenylalanine (4PH) in IP15 to aspartate (D) and glutamate (E) residues, respectively.

The peptide-bound ADGRG2 receptor was prepared and embedded in a phosphatidylcholine (POPC) lipid bilayer using the CHARMM-GUI online server [74,75,76,77,78,79]. The residues at the protein termini were assigned neutral patches (acetyl and methyl amide). The charged (NH3^+^ and COO^−^) peptide termini were left alone to mediate interactions with the surrounding ADGRG2 residues in the orthosteric pocket. The CHARMM36m [80] force-field parameters were utilized. CHARMM-GUI output files and scripts were used for GaMD simulations [79]. A total of 5000 steps of energy minimizations were carried out on the system, and a constant number, volume, and temperature (NVT) ensemble equilibration was performed for 125 ps at 310 K. Using an NPT ensemble, additional equilibration was carried out for 375 ps at 310 K. We then performed conventional MD (cMD) simulations on the systems for 10 ns at 1 atm pressure and 310 K temperature with the AMBER22 software package [81]. After the cMD runs, we performed GaMD equilibration for 64 ns for the ADGRG2–IP15-Gs complex system or 40 ns for the smaller ADGRG–IP15, ADGRG2–4PH-D, and ADGRG2–4PH-E complexes. This was followed by three independent GaMD production runs for 1200 ns for the ADGRG2–IP15-Gs system and 2000 ns for the rest of the simulation systems (ADGRG–IP15, ADGRG2–4PH-D, and ADGRG2–4PH-E). Long-range electrostatic interactions were computed with the particle-mesh Ewald summation method, and a cutoff distance of 12 Å was used for the short-range electrostatic and van der Waals interactions [82]. The ADGRG2–IP15-Gs system measures about 108 × 108 × 180 Å^3^ with ~246 lipid molecules, ~50,753 water molecules, and a total of 199,976 atoms. The other three systems (ADGRG–IP15, ADGRG2–4PH-D, and ADGRG2–4PH-E) measure 96 × 96 × 128 Å^3^ with ~205 lipid molecules, ~20,359 water molecules, and a total of 95,486, 93,929, and 93,332 atoms, respectively (Table 2).

All analyses were conducted on a GaMD trajectory using VMD and CPPTRAJ [83,84]. To identify the 20 most representative peptide conformations in the receptor binding pocket, hierarchical agglomerative clustering was conducted, focusing on the peptide RMSD in comparison to the initial computational model. The torsional angles of the rotameric toggle switch W^6.53^ of ADGRG2 were measured in each system using Bio3D [85]. Furthermore, the reweighted free-energy profiles were calculated by combining all simulation trajectories for each system using the PyReweighting toolkit[86]. The effect of residue interactions on ADGRG2 receptor deactivation was examined by investigating the residue–residue interactions formed between the peptides and the ADGRG2 receptor. Additionally, the distance between the intracellular ends of TM3 and TM6 of ADGRG2 was tracked over the course of the simulation. Peptide RMSD and receptor TM3–TM6 distances were measured using a bin size of 1 Å. For each of the peptide structural clusters, free-energy values were reweighted, and the cutoff was set to 500 frames in a bin. Finally, the most highly ranked structural clusters of the peptide in each system were evaluated and compared with the cryo-EM structure of the IP15-Gs-bound ADGRG2.

## Figures and Tables

**Table 1:** Summary of important residue interactions between the peptides and ADGRG2 along with the TM3–TM6 distances identified for each state of the simulation systems.

System	State	L^3.58^-R^6.40^ Distance (Å)	Peptide-Receptor Interactions	
			Hydrogen Bonding	Salt-Bridge
ADGRG2-IP15-Gs	Active	~18.7 Å	R617-N^5.32^, R617-I780^ECL2^, R617-W779^ECL2^, L615-Y^5.36^	I607-D^2.67^
ADGRG2-IP15	Intermediate	~14.7 Å	R617-N^5.32^, R617-I780^ECL2^, L612-W779^ECL2^, L615-Y^5.36^, I607-D^2.67^	I607-D^2.67^
ADGRG2-4PH-E	Inactive	~11.8 Å	L620-N781^ECL2^, S619-N781^ECL2^, R617- N^5.32^, R617-I780^ECL2^, L612-N^7.46^, S608- D^2.67^, I607-D776^ECL2^	_
ADGRG2-4PH-D	Inactive/bound	~11.1 Å	P621-Y766^ECL2^, P621- S765^ECL2^, P621-G767^ECL2^, T618-N^5.32^, D609-W^6.53^, D609-N^7.46^	I607-D^2.67^
	Inactive/lifted	~11.2 Å	P621-M^1.36^, R617-D776^ECL2^	I607-D^2.67^

**Table 2:** **Summary of the GaMD simulations performed on different systems of ADGRG2** ^(a)^. While IP15 is an agonist, the 4PH-E and 4PH-D are peptide antagonists of ADGRG2. ^(b)^*ΔV* is the harmonic boost potential that was added to the system to accelerate sampling of the potential energy surface. The average and the standard deviation of these boost potentials are reported in Table 2.

System ^(a)^	*N_atoms_*	Dimension (Å^3^)	cMD Runs (ns)	GaMD Equilibration (ns)	GaMD Production (ns)	*ΔV*^(b)^ (Kcal/mol)
ADGRG2-IP15-Gs	199,976	108 × 108 × 180	10	64	1200 ns × 3	14.42 ± 4.31
ADGRG2-IP15	95,486	96 × 96 × 128	10	40	2000 ns × 3	14.08 ± 4.23
ADGRG2-4PH-E	93,332	96 × 96 × 128	10	40	2000 ns × 3	13.85 ± 4.20
ADGRG2-4PH-D	93,929	96 × 96 × 128	10	40	2000 ns × 3	14.26 ± 4.27

## Data Availability

The data presented in this study are available in article and supplementary material.

## Supplementary Materials

The following supporting information can be downloaded at: https://www.mdpi.com/article/10.3390/molecules29010164/s1, Figure S1: The starting Computational Model. (a) Represent the computational model of ADGRG2-IP15-Gs, the membrane bilayer Phosphatidylcholine (POPC) were rendered as sticks, the ADGRG2 receptor with the Gs protein were rendered as cartoon while the sodium and chlorine ions were rendered as spheres. The water model used TIP3P was rendered as watermark and colored ice cube. (b) Represent the computational model for ADGRG2-IP15, ADGRG2-4PH-D, ADGRG2-4PH-E. Figure S2: The RMSD of the ADGRG2 peptide agonist and antagonists. (A–D) The time-course plot depicts the RMSD of the ADGRG2 peptides (agonist and antagonists) and its deviation from the starting structure. All simulations were carried out in triplicates. Figure S3: L3.58–R6.40 distance plot for ADGRG2 systems. (A–D) The distance plots depict the L3.58–R6.40 distance of ADGRG2-IP15-Gs, ADGRG2-IP15, ADGRG2-4PH-E, and ADGRG2-4PH-D systems respectively. All simulations are done in triplicates.
