# Supplementary material for: Mechanistic Insights into Peptide Binding and Deactivation of an Adhesion G Protein-Coupled Receptor
Source: Molecules. 2023 Dec 27;29(1):164. doi: 10.3390/molecules29010164 (PMC10780249; doi:10.3390/molecules29010164)
Supplement: Supplementary file 1 [file molecules-29-00164-s001.zip › molecules-2769469-supplementary.pdf]

ADGRG2 Supplementary Figures

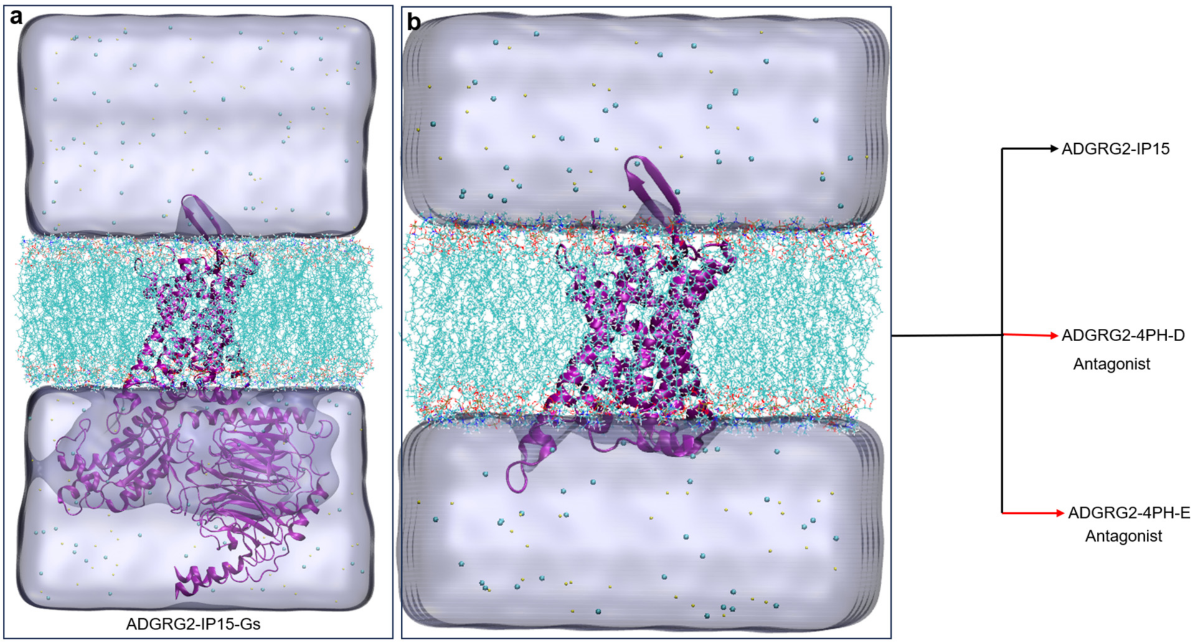

Figure S1: The starting Computational Model. a) Represent the computational model of ADGRG2-IP15-Gs, the membrane bilayer Phosphatidylcholine (POPC) were rendered as sticks, the ADGRG2 receptor with the Gs protein were rendered as cartoon while the sodium and chlorine ions were rendered as spheres. The water model used TIP3P was rendered as watermark and colored ice cube. **b)** Represent the computational model for ADGRG2-IP15, ADGRG2-4PH-D, ADGRG2-4PH-E.

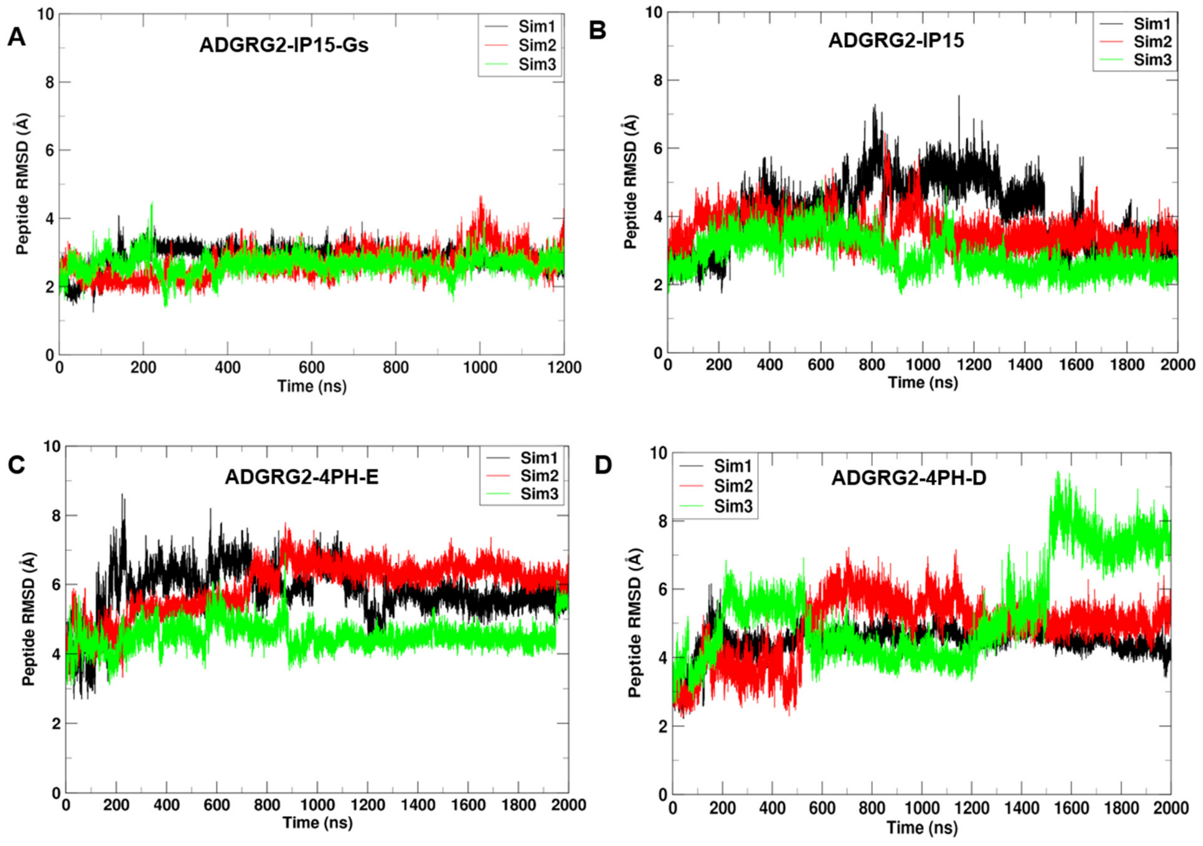

Figure S2: The RMSD of the ADGRG2 peptide agonist and antagonists. A – D) The time-course plot depicts the RMSD of the ADGRG2 peptides (agonist and antagonists) and its deviation from the starting structure. All simulations were carried out in triplicates.

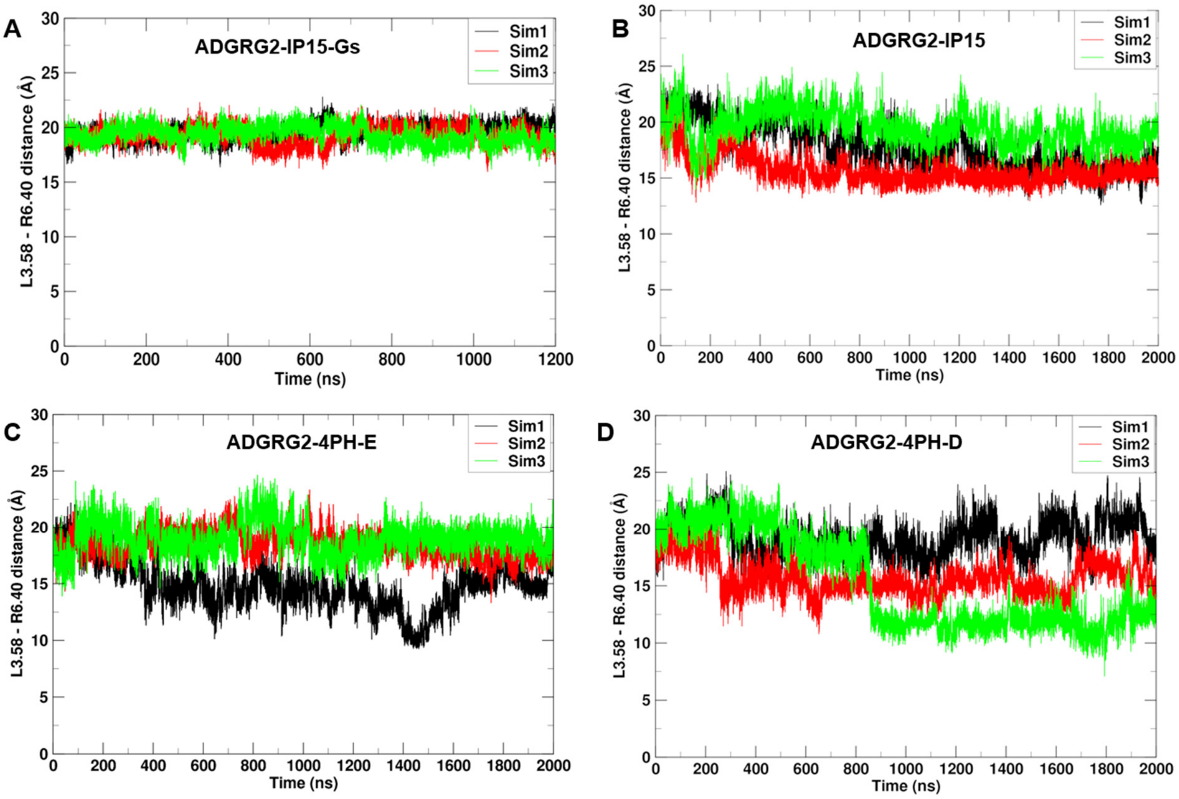

Figure S3: L3.58 – R6.40 distance plot for ADGRG2 systems. **A - D)** The distance plots depict the L3.58 – R6.40 distance of ADGRG2-IP15-Gs, ADGRG2-IP15, ADGRG2-4PH-E, and ADGRG2-4PH-D systems respectively. All simulations are done in triplicates.
